# “Knowing That I’m Not Necessarily Alone in My Struggles”: UK Autistic Performing Arts Professionals’ Experiences of a Mentoring Programme

**DOI:** 10.1007/s10803-021-05394-x

**Published:** 2021-12-29

**Authors:** Eleanor Buckley, Elizabeth Pellicano, Anna Remington

**Affiliations:** 1 UCL Centre for Research in Autism and Education (CRAE), University College London, London, UK; 2 Macquarie School of Education, Macquarie University, Sydney, Australia

**Keywords:** Autism, Employment, Mentoring, Support, Arts

## Abstract

This research examined whether professional mentoring could have a positive effect on the occupational self-efficacy of autistic performing arts professionals. We compared the outcomes of one group who received mentoring to a waitlist control group. 26 participants took part in this study: 15 autistic mentees and 11 mentors, three of whom were also autistic. The mentoring programme was well received and felt to be beneficial by the participating mentees and mentors, particularly regarding gains in mentees’ occupational self-efficacy. Professional mentoring also addressed several work-oriented challenges identified by autistic performing arts professionals such as feelings of isolation in the industry and need for consultation and advice on both a professional level, and for mentees with autistic mentors, also a neurodivergent one.

## Introduction

Autistic adults work, or seek work, across a variety of industries, yet many who wish to enter employment often struggle to do so (Lorenz et al., 2016; National Autistic Society, 2016; Roux et al., 2013). Autistic adults who are currently, or have been, employed report multiple challenges in their workplaces, ranging from difficulties with social communication and interactions, to tolerating unpredictable situations, or adapting when last-minute changes occur (Burt et al., 1991; Hurlbutt & Chalmers, 2004; Remington & Pellicano, 2018). These challenges are often due to negative interactions with, or attitudes of, employers and colleagues, as well as a lack of employment-based support (Baldwin et al., 2014; Buckley et al., 2020; Hurlbutt & Chalmers, 2004; López & Keenan, 2014; Lorenz et al., 2016; Unger, 2002).

While autistic people may encounter these challenges in every type of career, they may be exacerbated in certain professions. One such profession is the performing arts, an industry dominated by short-term contracts, with high reliance on networking and social interaction. Approximately one quarter of performing arts graduates work as freelancers, in comparison to around 5% of the general graduate population (Planit, 2020). Being employed in a project-based system, with frequent bidding for work, inherently involves a high level of uncertainty, and may undermine autistic people’s ability to achieve the sense of routine with which many feel comfortable. Furthermore, applying for and attending job interviews and auditions necessary to secure the next project are scenarios that require an adeptness with social interaction and communication, which autistic people can find challenging (American Psychiatric Association, 2013; Menger, 2006; VanBergeijk et al., 2008).

Creativity has not typically been associated with being autistic (Craig & Baron-Cohen, 1999). Yet, recent research has shown that autistic people have also been shown to excel at producing novel responses on creative tasks (Kasirer & Mashal, 2014; Best et al., 2015) and are working in creative industries such as the performing arts—a profession where autistic traits, including a high level of focus and ‘out-of-the-box’ thinking, may well be advantageous (Buckley et al., 2021). In our previous work with autistic performing arts professionals, however, they reported facing similar challenges to those described above by autistic employees outside this field. They also highlighted other challenges, including the industry’s emphasis on networking as a means to find and gain employment, their struggle to tolerate and adapt to an often changing or ill-suited working environment, and negative and ill-informed attitudes that they sometimes encountered from non-autistic co-workers (Buckley et al., 2020). Alongside describing the multitude of challenges that they face in their workplaces, autistic performing arts professionals also suggested the types of support that they felt would help them overcome these challenges. Many participants felt that having professional mentorship would be beneficial, particularly to help with networking, troubleshooting workplace concerns and guidance on career progression (Buckley et al., 2020).

Employment-focused mentoring for autistic adults is often suggested by researchers as a potentially effective strategy for support. While there have been some higher-education focused programmes (Lucas & James, 2018; Siew et al., 2017; Thompson et al., 2018), there is a scarcity of programmes designed specifically for workplace support (Gelbar et al., 2014). In one of the few existing studies, mentoring was trialled as a form of employment-based support for autistic adults from a range of backgrounds, as part of a broader curriculum that also included several hours per week of skill-building sessions and workplace exposure (Nicholas et al., 2018). The autistic mentees who took part (n = 14) reported an increase in skill acquisition, but there were no quantitative measures recorded and no specific outcomes were linked to the mentoring aspect of the programme (Nicholas et al., 2018). Another pilot study on a mentoring programme for autistic mentees (n = 12) examined changes in self-reported wellbeing using the Personal Wellbeing Index (Cummins et al., 2003) and analysed semi-structured interviews that took place with the mentees and mentors after the programme had finished (Martin et al., 2017). Following programme completion, the authors reported increases in mentees’ satisfaction with what they were achieving in life and satisfaction with life as a whole. Benefits were also reported by both mentees and mentors: the mentees felt that the mentoring was helpful in enabling them to progress toward self-identified goals, while the mentors also felt that they had met their own goals for taking part in the programme and reported gains in their self-confidence and knowledge around supporting autistic mentees.

This preliminary research is encouraging. Yet, such research has not addressed directly *why* mentoring might be beneficial for autistic employees. Research beyond the field of autism has shown that mentoring has a positive influence on occupational self-efficacy (Feldman et al., 2010; Jnah et al., 2015; St-Jean & Mathieu, 2015)—that is, the belief an individual has in their own ability to accomplish work-related tasks (Bandura, 1977)—and that such self-efficacy is in turn linked to workplace success and wellbeing (Bandura, 1977; Judge & Bono, 2001; Luszczynska et al., 2005). Social-cognitive theory (Bandura, 1986) suggests that mentoring might act on an individual’s sense of self-efficacy via role modelling and vicarious experience as well as social persuasion, especially through encouraging and providing feedback on mentee skills (cf. St-Jean & Mathieu, 2015).

Autistic adults without intellectual disability have been shown to have significantly lower self-efficacy in both general and occupational self-efficacy than neurotypical adults (Lorenz & Heinitz, 2014). Furthermore, self-efficacy has been shown to be better in workplaces that provide individualised support for autistic employees’ specific needs, in comparison to those that do not (Lorenz et al., 2016). Self-efficacy is also an important predictor of quality of life (Luszczynska et al., 2005; Nota et al., 2007; Shoji et al., 2015; Taylor et al., 2006; Vauth et al., 2007), which has been repeatedly shown to be poorer in autistic adults than in neurotypical people (Kamio et al., 2013; Kamp-Becker et al., 2010). Therefore, employment-based supports that target self-efficacy may be one important way both to improve career success and also positively affect quality of life in a population who often report difficulties in this area.

Mentoring might have a positive influence on autistic professionals’ self-efficacy for other reasons, too. Interactions between autistic and non-autistic people can often be challenging due to a lack of reciprocity and mutuality (Milton, 2012; Milton et al., 2017). Yet mentoring at its core involves a reciprocal relationship between the mentee and the mentor in which the mentor plays a critical role in deliberating the mentees’ personal and professional career-related dilemmas (Paul, 2009). It is a form of social support that enables the mentee better to see the challenges facing them and devise strategies to overcome them, potentially boosting their sense of self-efficacy in the process. Just like other forms of social support, trust and perceived similarity are important in building strong mentor–mentee relationships (Son & Kim, 2013; St-Jean & Mathieu, 2015). Having a trusted mentor that is in a similar situation—in this case, working in the performing arts profession—may enable the autistic mentees better to appreciate their own situation and devise responses to any challenges that are posed.

### The Current Study

Given the importance of the potential relationship between mentoring and self-efficacy, in the current study we examined autistic performing arts professionals’ experiences of a 10-week mentoring programme designed to improve their occupational self-efficacy. This programme was conducted within the context of a pilot, two-armed randomised controlled trial, in which autistic mentees were randomly assigned to the modification (mentoring) group and a waitlist control group.

Our aims were twofold. First, we sought to determine whether our mentoring programme could be implemented successfully and be acceptable to participants. Second, we examined from the perspectives of mentors and mentees whether the mentoring programme was perceived to enhance occupational self-efficacy and, if so, in what ways.

## Method

### Participants

In total, 26 participants took part in this study: 15 mentees (five female, seven male, three non-binary or other) and 11 mentors (six female, five male) (see Table 1). Mentees were required to: (1) be over 18; (2) self-identify as autistic; (3) be working or trying to work (full-time, part-time, or casual positions) in the performing arts; and (4) be based in the UK at the time of participation. All 15 mentees self-identified as autistic, with 12 having received an independent clinical diagnosis of an autism spectrum condition according to DSM-IV or DSM-5 criteria (American Psychiatric Association, 1994, 2013). Two were in the process of obtaining a diagnosis (and went on to receive their autism diagnoses after completion of the study), and one self-identified without a formal diagnosis. We included individuals who self-identified as autistic but had not yet received a formal diagnosis because adult diagnostic services are limited and those that do exist can have lengthy waiting lists and be financially costly (Unigwe et al., 2017). Eleven of the mentees had received diagnoses of one or more co-occurring conditions, including anxiety (n = 9) and depression (n = 9). The mentees reported a range of experience with performing arts, from under 1 year to 20 years (Median = 4 years). They were working, or interested in working, in different roles such as performing, writing, directing, and stage-managing. None of the mentees received other mentorship while taking part in the programme but four mentees (two in the modification group and two in the control group) reported receiving other types of support, such as financial, during the study period.

**Table 1:** Characteristics of mentees and mentors

	Modification group mentees n = 8	Waitlist control group mentees n = 7	Mentors n = 11
Age			
Mean (SD), years	34 (12)	31 (7)	41 (13.3)
Median, years	31	28	37
Range, years	19–54	24–42	27–63
Gender			
Female (including transgender female)	3	2	6
Male (including transgender male)	4	3	5
Non-binary or other	1	2	–
Ethnicity			
White	6	7	11
Black	1	–	–
Mixed	1	–	–
Self-identified as autistic (incl those undergoing autism assessment at time of study)	2	1	–
Clinical autism diagnosis	6	6	3
Co-occurring conditions			
Anxiety	4	5	N/A
ADHD	–	–	N/A
BPD	–	1	N/A
Depression	5	4	N/A
Dyslexia	1	–	N/A
Dyspraxia	1	–	N/A
OCD	1	1	N/A
Median years in performing arts (range)	5 (1–20)	4 (1–12)	10 (6–35)

Mentors were required to: (1) be over 18 years old; (2) have worked in the performing arts for a minimum of 5 years, indicative of sustained careers in the industry; and (3) be based in the UK at the time of participation. The mentors had varied roles within the performing arts, with many working in several roles across their careers, such as theatre company directors, actors, dancers, and writers. They also had been working in the performing arts for varying lengths of time, ranging from six to 35 years, with a median of 10 years’ experience. Three of the mentors themselves had received clinical diagnoses of autism.

Mentees were initially asked to report on which career-related topics they would like to receive mentorship. These topics included but were not limited to: applying and preparing for jobs/auditions; managing workplace relationships; applying for funding and writing about your work; networking and building professional partnerships; devising/developing new works; self-promotion and raising your professional profile; self-organizing and time-management; and advocating for access needs. Mentors were then selected for the programme and matched (by the first author) with mentees based on the overlap of their skillsets and expertise with mentees’ desired mentoring topics.

### Recruitment

Between September 2018 and November 2018, mentees and mentors were recruited through word-of-mouth and online advertisement using social media and UK community contacts. Mentors were asked to apply with their curriculum vitae and to provide information concerning their areas of expertise and topics they felt able to advise on in a mentorship role. Of the 23 submissions, 11 mentors were selected based on their skillsets matching the self-reported needs of at least one mentee. Each mentee in the modification group (n = 8) was mentored by a different mentor. Three mentors who had worked with the modification group also went on to mentor control group mentees (n = 7). In the control group, one mentor mentored two mentees, all other mentors worked with one mentee. Five of the mentees were mentored by the three autistic mentors, three in the modification group and two in the waitlist control group, the remaining ten mentees were mentored by non-autistic mentors. Mentors were compensated for their time at industry rates.

### Mentoring Programme

The mentoring programme was designed to improve occupational self-efficacy in autistic performing arts professionals. The programme consisted of mentees and mentors meeting remotely over video/audio/text-based chat or phone for a one-hour mentoring session once every 2 weeks for 10 weeks, completing 6 sessions in total. The mentees and mentors were encouraged to keep in email contact between sessions in order to schedule further mentoring sessions and follow up on discussions. Participants were asked not to schedule any extra sessions, to limit communication to email outside of sessions, and not to physically meet while taking part in the programme. The content of the mentoring sessions was decided between each mentor and their mentee but was focused on career-based topics (see Participants section).

**Fig. 1:**
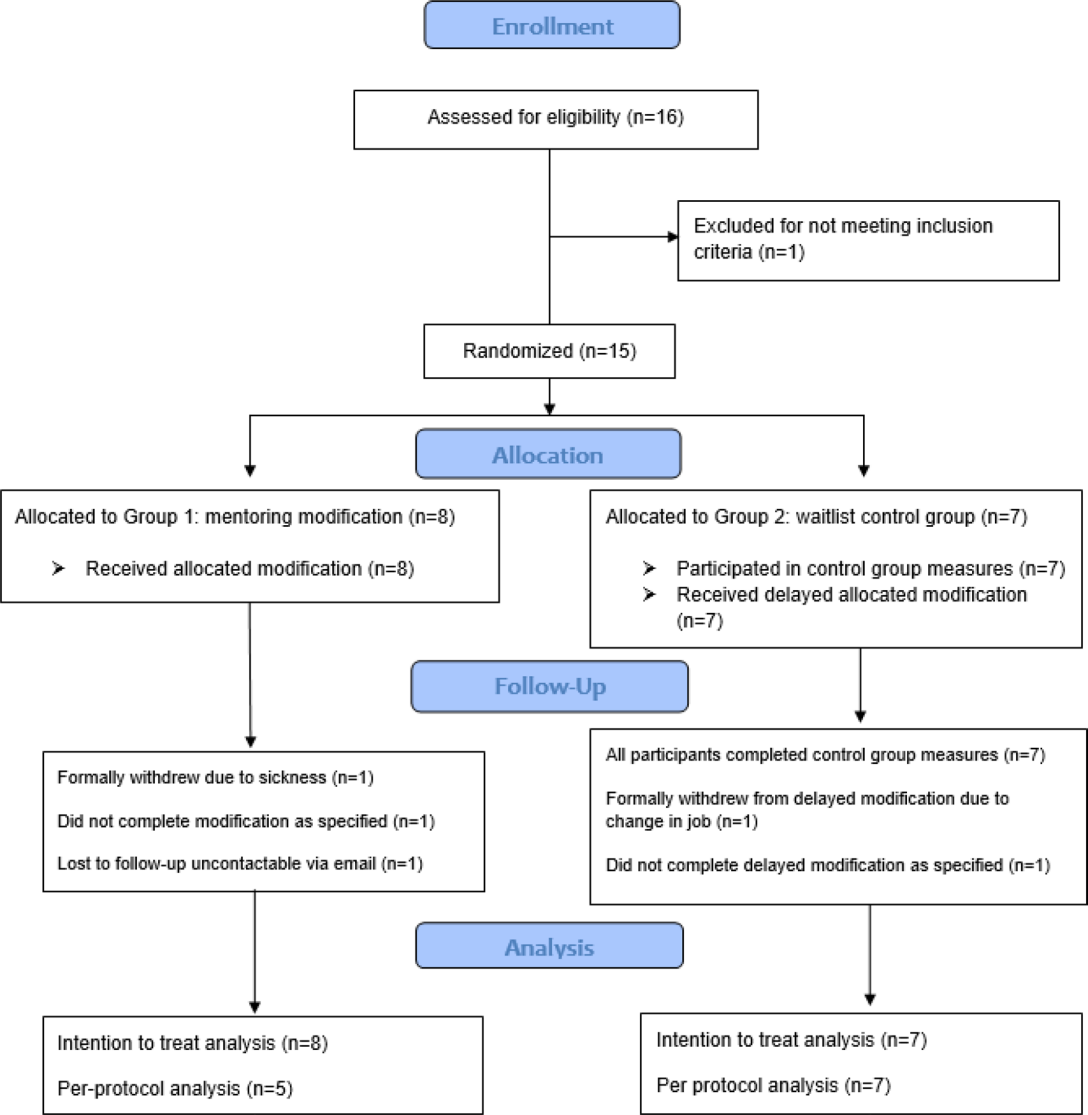
Flow of participants through trial. The two mentees who did not complete the modification/delayed modification as specified completed all six mentoring sessions but did not complete within the specified timeframe of 8–12 weeks

Prior to commencing the programme, all mentors attended mandatory *autism and the workplace* training co-designed and co-led by the first author and an autistic colleague with an arts background. The training comprised teaching the mentors about characteristics of autism and how these might contribute to challenges and strengths in the workplace; detailed instruction concerning the structure and aims of the programme; possible strategies to use when supporting autistic people through mentorship; and the opportunity to ask questions about any aspect of the programme and details on how to access support for themselves or their mentees while participating in the programme. Waitlist-control group participants began by continuing with their usual working lives and any other support they were accessing. Approximately 4 weeks after the modification group completed their programme, the control group then received the same mentoring of six sessions delivered across 10 weeks. Figure 1 shows the flow of participants through the trial.

### Measures

The programme was designed to determine whether occupational self-efficacy could be improved through receiving professional mentorship. Both quantitative and qualitative methods were used to address this question. Mentees completed questionnaire measures of occupational self-efficacy (our primary outcome of interest) and quality of life (secondary outcome) at baseline (0 weeks), post-modification (11 weeks) and follow-up (26 weeks). Critically, we also conducted in-depth semi-structured interviews to elicit mentees—including those from the modification and waitlist control groups—and mentors’ views and experiences of taking part in the mentoring programme.

### Quantitative Measures

To measure *occupational self-efficacy,* we created a bespoke scale based on Bandura (2006) but adapted specifically to target professionals’ perceived confidence when performing activities associated with their performing arts careers (Bennett, 2009). This scale, previously administered to a large sample (n = 1427) of performing arts professionals, showed excellent internal consistency (Cronbach’s α = 0.94) (Buckley et al., 2021). The self-efficacy scale contained 24 statements to which participants could respond to each item with a score ranging from 0 (“not at all confident”) to 10 (“extremely confident”). Items used included, for example, “interview / audition for roles”, “fully understand all instructions given to me”, and “get a colleague or peer to help me if I have difficulty interacting with others at my workplace”. Item scores were averaged to yield a mean self-efficacy score. Higher scores reflect greater occupational self-efficacy. The scale in the current sample also showed excellent internal consistency (Cronbach’s α = 0.95).

To measure *quality of life,* we used the World Health Organization abbreviated version of the WHOQOL-100 quality of life assessment (WHOQOL-BREF; The Whoqol Group, 1998), including the additional autism-specific items (ASQoL) developed by McConachie et al. (2018) (total of 35 items). The four WHOQOL-BREF domains have acceptable internal consistency (αs ≥ 0.7; Skevington et al., 2004) and the ASQoL has good internal consistency (α = 0.82; McConachie et al., 2018). The WHOQOL-BREF contains 26 items (e.g., “how satisfied are you with your ability to perform your daily living activities?”), which measure four domains of quality of life (physical, psychological, social, environment). Each domain is scored separately. The ASQoL contains eight items that produce a total score (e.g., “do sensory issues in the environment make it difficult to do things you want to do? For example, supermarket too noisy, public transport too busy, etc.”) and one global item about autistic identity (e.g., “Are you at ease (OK) with ‘Autism’ as an aspect of your identity?”). Higher scores on the four domains of the WHOQOL-BREF (in the current study: physical domain α = 0.82; psychological domain α = 0.83; social domain α = 0.69; environment domain α = 0.84) and the ASQoL add-on module (in the current study α = 0.79) reflect greater quality of life within those specific areas.

Mentees were asked to complete these measures at the beginning (week 0) and end of the modification (week 11), as well as the 3-month follow-up (week 26).

### Qualitative Measures

Semi-structured interviews were conducted with all participants prior to the beginning of the mentoring programme (1–14 days before week 0) and again once it was completed (week 11–12). Interviews were recorded with participants’ prior consent and professionally transcribed verbatim. In the pre-mentoring interviews, mentees and mentors were asked about their hopes and expectations around taking part in the mentoring. In the post-mentoring interviews, mentees and mentors were asked about their experiences, the perceived impact of the programme and any challenges and/or benefits to taking part. See Supplementary Information for full interview schedules.

### Procedure

This research study received ethical approval and was run in accordance with the ethical standards of UCL Research Ethics Committee and with the 1964 Helsinki Declaration and its later amendments. All participants provided written informed consent prior to participating in this study.

Once included in the study, mentees were randomly assigned to modification vs. waitlist control group using a block randomization method. Both modification group and waitlist control group mentees completed occupational self-efficacy and quality of life measures 1–14 days before the mentoring programme in December 2018. Quantitative outcomes were not examined for waitlist control group mentees receiving the delayed mentoring. All participants (mentors and mentees in both groups) completed individual semi-structured interviews over the phone, on video-call, or in-person, either on University premises or in a location of their choosing within 2 weeks of beginning their first mentoring session. This meant interviews took place across different time periods for the modification and waitlist control groups (December 2018 and March 2019, respectively). Pre-mentoring interviews with mentees ranged in length from four to nineteen minutes (Median = 6 min), and with mentors five to fifteen minutes (Median = 9 min). Mentees and mentors were then introduced to each other over email by the first author and asked to schedule their six mentoring sessions with each other, with the aim of having a mentoring session every 14 days on average (aiming for all six sessions to be completed in 10 weeks; upper and lower bounds of acceptable completion of the 6 sessions = 8 weeks to 12 weeks). Mentees and mentors were asked to complete online questionnaires after each mentoring session in which they were asked to describe briefly the content and their thoughts on the session.

Within 2 weeks following the final mentoring session, mentees and mentors were interviewed again about their experiences. Interviews were conducted with all but one of the mentees (who had withdrawn from the study due to illness; see Fig. 1). All mentors took part in post-mentoring interviews. Mentee and mentor interviews from those who were unable to complete the modification were still included, where possible, to better understand the challenges that had led to these circumstances. Post-mentoring interviews with mentees ranged in length from 15 to 29 min (Median = 21 min), and for mentors 18 to 36 min (Median = 24 min).

### Data Analysis

#### Quantitative Analysis

Pre- and post-modification questionnaire data were analysed to assess any change in occupational self-efficacy (primary outcome) and quality of life (secondary outcome) measures. Given the small sample size, we examined changes in scores for each of the dependent variables (occupational self-efficacy; WHOQOL-BREF domains 1–4; ASQoL Total score) using a Reliable Change Index (RCI; Jacobson & Truax, 1991) computed by dividing the difference between the pre- and post-mentoring scores by the standard error of the difference between the two scores. The RCI indicates whether an individual’s change in scores over time is considered statistically significant.

#### Qualitative Analysis

Qualitative data from pre- and post-mentoring interviews with all participants were analysed using reflexive thematic analysis (Braun & Clarke, 2006, 2019). The transcripts were analysed using an inductive (bottom-up) approach where themes were created within a ‘contextualist’ method of critical realism (Willig, 1999). The first and last authors carried out the thematic analysis and approached the analysis from the perspectives of psychology researchers who do not identify as autistic, and therefore analysed the data from the perspective of outside interpreters. Data were initially coded by the first author without any pre-existing coding schemes, and surface-level themes were identified. Themes for each participant group were first generated separately and then merged across participant groups to determine areas of similarity and incongruity, in order to provide a multi-informant view of the mentoring. The analysis was reflexive, meaning that the authors moved backwards and forwards between the data and analysis. The authors met together several times to discuss the themes and subthemes, ensuring that the themes and their definitions encompassed the patterns of shared meanings across the entire data set.

## Results

### Quantitative Results

#### Mentee Characteristics

Of the 16 mentees assessed for eligibility, 15 met the inclusion criteria (one did not identify as autistic). Mentees were randomised to the modification (n = 8) or waitlist control (n = 7). During the modification, two mentees were not able to complete the modification as specified: one withdrew due to sickness and one did not complete the modification in the timeframe specified. One additional mentee completed the modification but did not participate in 3-month post-modification follow-up measures. All participants were included in the intention-to-treat analysis (see Fig. 1).

The demographic data (Table 2) suggested that the modification and waitlist control groups were similar in terms of distributions of age, gender, years in the arts, and participants who were receiving other support. The groups were too small to run sufficiently powered statistical comparisons.

**Table 2:** Participant measures on outcome variables at pre-intervention, post-intervention, and 3-month follow-up

Measure	Group	Pre-intervention 0 weeks modification group n = 6 control group n = 7			Post-intervention 11 weeks modification group n = 6 control group n = 7			Follow-up 26 weeks modification group n = 5		
		M	SD	Range	M	SD	Range	M	SD	Range
Occupational self-efficacy	Modification group	6.1	2.4	2.7–8.8	8.1	0.9	6.7–8.9	8.2	1.2	6.5–9.6
	Control group	4.9	1.8	1.5–6.8	5.1	1.9	1.7–6.8	N/A	N/A	N/A
WHOQOL-BREF Physical domain	Modification group	13.3	4.2	7–18	15.5	2.2	13–19	16.0	2.5	12–18
	Control group	13.0	1.4	11–15	13.3	2.2	10–17	N/A	N/A	N/A
WHOQOL-BREF Psychological domain	Modification group	12.7	4.0	5–17	15.5	1.8	13–18	15.2	2.2	13–18
	Control group	11.0	1.2	10–13	12.0	1.5	11–15	N/A	N/A	N/A
WHOQOL-BREF Social domain	Modification group	11.8	5.4	5–20	15.3	3.6	9–20	13.6	4.2	9–20
	Control group	13.3	3.0	8–16	13.0	2.9	8–15	N/A	N/A	N/A
WHOQOL-BREF Environment domain	Modification group	12.5	4.0	6–18	15.8	2.6	12–19	15.2	3.2	11–20
	Control group	12.4	1.5	10–14	13.6	2.4	11–18	N/A	N/A	N/A
ASQoL Total	Modification group	3.0	1.3	1.4–4.5	3.7	0.7	2.8–4.5	3.6	0.8	3–5
	Control group	2.8	0.5	2.4–3.9	3.1	0.4	2.5–3.8	N/A	N/A	N/A
ASQoL Global	Modification group	4.3	1.2	2–5	4.7	0.8	3–5	4.6	0.9	3–5
	Control group	3.9	1.1	2–5	3.7	1.1	3–5	N/A	N/A	N/A

#### Analyses

**Fig. 2:**
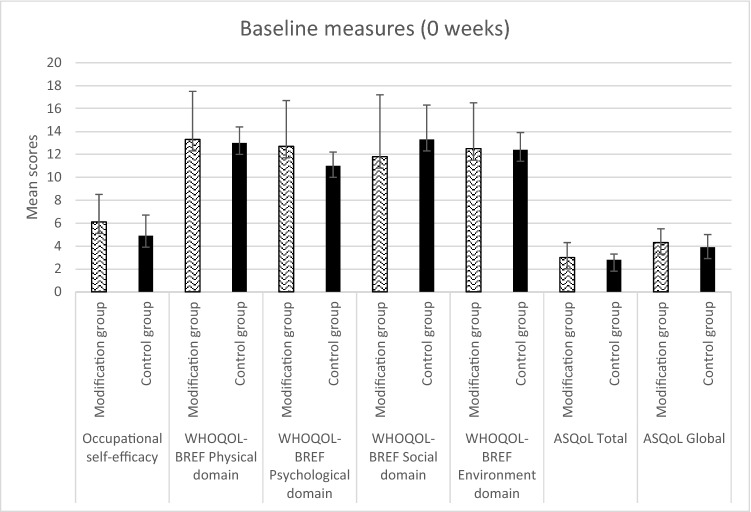
Comparison of mean scores on all measures between modification and control group mentees at baseline. Standard deviation values are shown using error bars

**Fig. 3:**
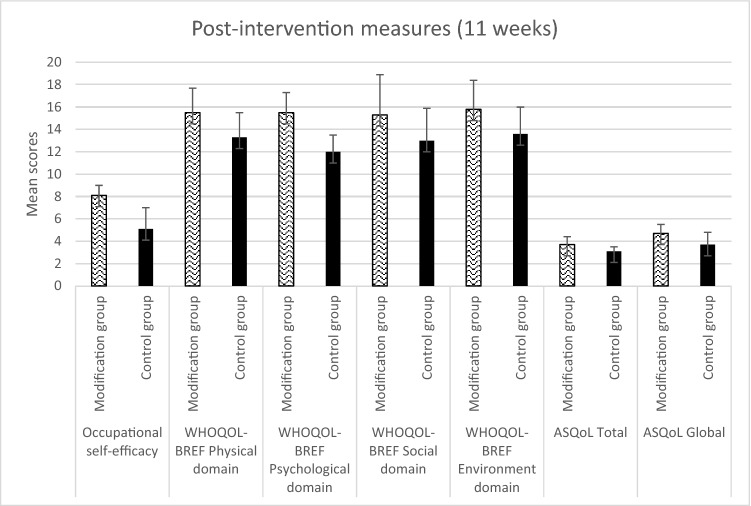
Comparison of mean scores on all measures between modification and control group mentees at post-modification. Standard deviation values are shown using error bars

Table 2 summarises the results from the comparison of outcome measures taken at each time-point. Six of the eight modification group mentees completed pre-modification and post-modification measures (occupational self-efficacy and quality of life), and all seven of the waitlist-control group mentees completed the same measures. Five of the modification group mentees went on to complete the same measures at 3-month follow-up (see Table 2), but the control group did not as they had started to receive their delayed mentoring programme. See Fig. 2 and 3 for graphs showing the mean scores on all measures for each group.

For mentees in the modification group, while absolute scores on all measures increased at post-modification testing compared to baseline, reliable changes in scores (as measured by the RCI) were only seen in some of the mentees. Reliable changes in occupational self-efficacy score were observed in four out of the six modification group mentees, such that their self-efficacy score was higher after participating in the mentoring programme than at baseline. Significant reliable changes were not observed in the majority of modification group mentees with regard to quality-of-life scores. One mentee reported significant increases in the physical, psychological, and environment domains after having received the mentoring. One mentee reported significant positive change in the social domain, and one other mentee reported significant positive change in the environment domain. These results indicate the modification significantly improved occupational self-efficacy and quality of life in some of the autistic mentees. Scores stayed relatively stable on all measures between post-modification testing and 3-month follow-up, with no reliable change found in scores. Waitlist control participants did not see any reliable changes in scores between baseline and post-modification (see Table 2).

### Qualitative Results

Themes and subthemes from the pre- and post-mentoring interviews with all of the mentees (n = 15) and mentors (n = 11) are presented in turn below. As we identified similar themes across the various groups at each time point, we report the themes from all groups together here. All themes and subthemes (italicised in the text) and example quotations are listed in Tables 3 and 4.

**Table 3:** Themes and subthemes from pre-mentoring interviews

Themes	Subthemes	Quotations
Practical concerns	Identifying a schedule that works	“the reality of my life as a single mother of three and trying to make a living as a performing artist, some of that stuff gets in the way” [Mentee E]
		“I work like 5 days a week, so it would just be … just … well, timing our mentoring sessions right” [Mentee F]
		“I’m a disabled person myself I think working around both our access requirements will be interesting but not necessarily a challenge” [Mentor U]
	Reaching shared understanding	“Being able to say what I want to say I find difficult to get the words across. To make people understand what I’m trying to say” [Mentee D]
		“I'm anticipating that there will be some issues around clarity, maybe, of what they want and how I can help them” [Mentor X]
		“Effectively communicating what the issues are” [Mentee Q]
Anxious about so many unknowns	Apprehensive about the unspecified aspects of the mentoring	“Just being nervous about not knowing who and speaking to and what they’ll be like, just the unknown of it all” [Mentee D]
		“I am nervous, I am … I am very … I get … I am … I don’t know what the challenges are going to be” [Mentee R]
		“There is definitely a big unknown question mark at this point about what that person is going to need” [Mentor W]
	So much depends on the strength of the relationship	“I’m apprehensive that I like won’t be able to like establish a good relationship with the mentee and that we won’t find a good way of talking” [Mentor T]
		“It will rely completely on the relationship with the other person” [Mentee U]
		“I think it will probably take a while to work out the best way of working together” [Mentor V]
	Will it be a positive experience?	“I would worry that their experience of it wasn’t positive, just generally positive. And maybe that has to do with like lack of communication if the person stops making contact that I might feel well because I’m not doing a very good job or just not being able to pitch it right” [Mentor Y]
		“I just wouldn’t be useful and that the mentee would find it … that they maybe would want to opt out after a few sessions” [Mentor S]
A place to share and learn	A chance to feel less isolated	“Getting advice of going, “Okay, I’m not the only one going through this”; just to find out that there is still a hand out kind of going, “Yeah, we’re all going through this together”” [Mentee W]
		“I think it will provide me with purpose, it will provide me with knowing there are people out there like me. That’s really important” [Mentor Z]
		“I like the fact that the programme was looking at ASD and autism and that you can talk to someone who, you know, has had similar challenges or experiences and I think that will be really very nice for me because you don’t always get that opportunity” [Mentee N]
	Increase autism knowledge	“It will make me think about myself in the industry more, I also think it’s a really brilliant opportunity to gain skills in working with autistic people and working with them in the arts” [Mentor Y]
		“It’ll help if I’m working with any other people with autism in the future” [Mentor P]
		“I think this will be really beneficial for me to understand how the industry can be more accessible to autistic professionals” [Mentor W]
Hopeful for long-term benefits	Increased self-belief	“What this mentoring could do for me is that I can … this can give me the confidence to build up myself and then say okay let’s see what … let’s see how we go down this route” [Mentee L]
		“It’s building my confidence as a mentor as well if I see that I have really helped someone and they’re really happy with it and it helps them go further in their career” [Mentor B]
		“I hope it’s going to make me more confident to do this kind of thing more often because it’s something that I’ve been planning to do for a long time” [Mentor Q]
		“Increased confidence and feeling like it is my right to try to do these things and access these spaces” [Mentee U]
	Mapping out career strategies	“Giving me some necessary skills and advice as to how to improve my own career from where it’s at the moment “ [Mentee K]
		“Would find a little bit more of a pathway for myself and a little bit more of a strategy” [Mentee J]
		“It’s also for them to sow seeds in you so that you can better mentor other people after and for you to sow seeds in them that might blossom a year, 2 years down the line” [Mentor Z]

**Table 4:** Themes and subthemes from post-mentoring interviews

Themes	Subthemes	Quotations
A need for flexibility	One structure doesn’t suit all	“I think 2 weeks is a great amount of time to be able to not only think about the previous session we’d talked about but also gear up to the next session and the work you’ve developed going into that next session” [Mentee I]
		“I would’ve liked is to be able to ration the sessions over a period of weeks or months” [Mentee O]
		“I honestly think the video chat was probably best because meeting face to face would’ve caused so many sensory difficulties and so much exhaustion from doing that I wouldn’t have got the same out of it. So, it was actually really convenient” [Mentee E]
		“I think the fact we were only able to communicate over Skype or phone was a problem. I think it’s different whenever you’re with someone in person” [Mentee A]
		“I found that amount of time to be pretty good. It meant that I only had to schedule an hour for the meeting but that was long enough to talk about stuff” [Mentee H]
		“It was difficult for them to engage for the whole hour, so we would often do half an hour to 40 min and then have other tasks that we would agree for the last 20 min” [Mentor S]
	Support needs to be implemented at the right time	“I think I would possibly wait… if I had a chance to start at whatever time I wanted I think I’d possibly wait until I was attempting to make a show or attempting to put on a show somewhere because then I could get advice on how to find a venue and funding and stuff, which is not advice that I think can be given hypothetically” [Mentee H]
		“I think it was a good idea in principle. I think [my mentee] and I had difficulties in that they just weren’t ready to plan or to work on anything, so that was kind of hard” [Mentor U]
	Being accommodating can be challenging	“I’m really up for being flexible, but I think I really tried to… like I think I really inconvenienced myself a few times because I was trying to just work with their schedule, so I think I probably could’ve been a bit more, “Yeah, we can rearrange but I can do this time”, rather than, “Yeah, sure, I can do four o’clock; I’ll make it work”, kind of thing” [Mentor T]
		“I found the kind of last minute cancellations and trying to rearrange things just frustrating” [Mentor X]
Good communication is key for managing expectations		“Be a bit more aware of how much is being put on each other’s plate and enforce that only so many things should be discussed, have clearer set of … be clearer with each other about how much communication’s going to be had because the mentor was trying to get more out of me than I was able to give both in time and mental health wise” [Mentee C]
		“I think it worked well in terms of communication because it was always very, you know, we’ll speak on Skype on this day at this time and I knew what to expect and it was structured so we knew what we were going to be talking about and what the goals were so having the goals and the structure made me able to kind of follow the process if you know what I mean without getting anxious” [Mentee N]
A confidence boost for many	Not defining success by other people’s standards	“To value myself because I’m me rather than place the values of others on myself if that makes sense, so stop like … to just say that I’m enough kind of thing” [Mentee B]
		“So it’s very much not the case of needing 100% from somebody to give me the thumbs up, but rather it’s for me to give myself the thumbs up” [Mentee I]
		“Really helped me focus on putting myself at the heart of my work, which was a journey that I’d sort of started – it was like an idea – but I think the mentoring really embedded that and gave me the confidence to say, “Actually, my experience is valid”” [Mentee E]
	Reflecting on achievements	“Being able to remind me about how effective these efforts I’m doing currently because with a lot of this kind of work you're sort of shouting into a vacuum and you don’t get much feedback until something clicks and so to be told, or at least to sort of realise that the stuff that you’re doing is actually proactive and positive is a helpful step in itself” [Mentee M]
		“You recognise achievements [together] that they’ve made, which they made a whole load in the time that we spoke together” [Mentor R]
	Opening the door to new opportunities	“I’ve started to network and I’m like confident enough to go on my own and everything which was a goal” [Mentee N]
		“I ended up submitting a play for [a playwriting prize] which I … I suppose I wanted to do but didn’t necessarily believe I would and it happened” [Mentee J]
Fostering an empathetic space	A safe and supportive environment	“It seemed like there was a space that he could actually be really truthful about the things that he does actually genuinely struggle with” [Mentor S]
		“It was just really, really lovely to have someone with that, you know, that level of experience to talk these things through with and be encouraged by” [Mentee J]
	Feeling less alone	“I think that the reduction in my anxiety and the feeling of being less alone is the most important” [Mentee N]
		“It’s been reassuring really, you know, just knowing that … knowing that I’m not necessarily alone in my struggles” [Mentee K]
	A mentor with lived experience is highly valuable	“They felt they could talk about a lot of stuff because I’m autistic and they’re autistic that they probably wouldn’t have raised if I wasn’t because when you’re scared of saying, “Oh I can’t, you know, I can’t ring them up,” you know, I probably wouldn’t tell a non-autistic person that, so there was a bit more openness I feel” [Mentor Z]
		“A benefit of having an autist Mentor: they’d been through it and understood and had dealt with all that stuff themselves” [Mentee E]
		“In other similar sessions that I’ve done I’m essentially having to explain the problems that exist more than actually taking advantage of the mentoring because people who are mentoring me have no idea of the barriers that exist for me” [Mentee M]
	The knowledge exchange could go further	“I think it might have been useful to have a chat sort of halfway through the mentoring sessions with other mentors just to see how they’re managing that balance” [Mentor V]
		“Something like establishing a network of mentors and mentees but how that would look I have no idea at the moment but that might be an interesting thing for people to exchange sort of insights that they want to share if that’s even an option” [Mentor Q]
A mutual learning opportunity	New knowledge	“Time management and organisation: with the things that we’d spoken about and the techniques that had been shared with me I thought, “I’ve got a better understanding of this now”” [Mentee F]
		“I learnt quite a lot about breaking things down. I guess what I was asked to do in that process quite a lot was use my experience and explain my take on something, and I was trying to do in as clear a way as possible. And so I think it definitely helped me to understand the things I know better” [Mentor T]
		“I found it really beneficial for my own professional artistic output in terms of, you know, they always say that teaching is the best way to learn” [Mentor Y]
	Increased autism knowledge for mentors	“They’re not an expert in autism so I think the benefits that they got is that they spoke to an actually autistic artist…So, in terms of education about autism I think that was very good because now they can go away and they’ll go, “Oh yeah, I understand a bit more now about autism and that it’s a spectrum”” [Mentee G]
		“I feel l have been a lot more prepared from this mentorship programme to then go into working with autistic creatives” [Mentor W]
	A constructive experience	“It’s been great. Like I say, it’s really been transformative; more so than any other personal development I’ve done and I’ve done a lot over the years” [Mentee E]
		“It’s just been really great to have this over this period of time. It’s got me focusing on really positive things, I’ve learned a lot from it, a huge amount. So yeah, I mean for me it’s been a very positive experience” [Mentee J]
The relationship can make or break the support	A clash of personalities	“We both reacted and didn’t really do anything to … positively progress those emotions we were feeling” [Mentee C]
		“This phase started off a little bit more challenging just because of personalities as in mind-sets. It was a little bit more of a challenge than in the last one to begin with but I think the results speak for themselves” [Mentor Q]
	Strong bonds can lead to success	“Just really easy, like [my mentor] is very easy to get along with, really personable and kind and, you know, you could tell that they wanted the best, like they were thinking about my best interest so that was very helpful” [Mentee N]
		“I felt like I built up a really good relationship with [my mentee] and we had a lot to talk about” [Mentor T]

Overall, prior to starting the mentoring, mentees and mentors were apprehensive about all of the unknowns concerning taking part in the programme, but also looking forward to the opportunity to focus on their goals and hopeful for long-term benefits. In post-mentoring interviews, the mentors and mentees reflected on how the programme had provided a useful learning opportunity and a confidence boost for many involved, although they also acknowledged the practical and emotional challenges involved in taking part in the programme.

### Pre-Mentoring Interviews

#### A Place to Share and Learn

All participants were invested in the idea that the mentoring programme would provide an opportunity to develop their career-based skills and be a space to share experiences openly. Many mentees and also some mentors expressed excitement that the mentoring would provide them with *a chance to feel less isolated*. They reported that the performing arts industry can be a lonely and difficult environment for many, and even more so for those navigating it with a disability: “Getting advice of going, ‘Okay, I’m not the only one going through this’; just to find out that there is still a handout kind of going, ‘Yeah, we’re all going through this together’” [Mentee C]. Some mentors also looked forward to *increasing their autism knowledge*. Several non-autistic mentors had not worked previously with autistic people in a mentoring capacity and therefore saw this as an opportunity to broaden their experiences and learn how to potentially adapt their own practices to be more inclusive: “It will make me think about myself in the industry more. I also think it’s a really brilliant opportunity to gain skills in working with autistic people and working with them in the arts” [Mentor Y].

#### Anxious About So Many Unknowns

Although the mentoring programme was structured, there were many elements to it that could not be predicted, such as the exact content of the sessions and whether the mentees and mentors would connect with each other. While participants taking part in the mentoring programme were excited about the opportunity, both mentees and mentors were also *apprehensive about the unspecified aspects of the mentoring* such as challenges that may arise over the course of the programme or who they were going to be paired with: “Just being nervous about not knowing who and speaking to and what they’ll be like, just the unknown of it all” [Mentee D]. Both mentees and mentors identified that the success of the mentoring programme relied on *the strength of the relationship* they would form with their mentoring partner, many were anxious yet hopeful about the bonds they would form: “I’m apprehensive that I like won’t be able to, like, establish a good relationship with the mentee and that we won’t find a good way of talking” [Mentor T]. Mentors also worried about whether they would be able to provide a useful and *positive experience* for their mentees: “I just wouldn’t be useful and that the mentee would find it … that they maybe would want to opt out after a few sessions” [Mentor S].

#### Practical Concerns

Mentees and mentors also discussed several challenges that they expected to arise during the programme. One potential difficulty was fitting the programme around their work and personal lives, as well as any access needs, and so *identifying a schedule that works* for both parties was important: “I work like 5 days a week, so it would just, well, timing our mentoring sessions right” [Mentee F]. Both mentees and mentors highlighted the importance of *reaching shared understanding* within the mentoring partnerships as to what the mentees wanted to achieve from the mentoring programme, and were expecting that there may be some challenges in effectively communicating and understand those desires: “I’m anticipating that there will be some issues around clarity, maybe, of what they want and how I can help them” [Mentor X].

#### Hopeful for Long-Term Benefits

All participants signed up to the programme with the expectation that it would be immediately useful to them, but many also hoped for more enduring changes to help them progress further in their careers. Both mentees and mentors were looking forward to seeing how the mentoring might *increase their self-belief* and build their confidence, hoping for “increased confidence and feeling like it is my right to try to do these things and access these spaces” [Mentee A]. Another potential benefit of the scheme recognised by the mentees and the mentors was the possibility to *map out career strategies* and learn skills that they could take forward with them in their professional lives: “Giving me some necessary skills and advice as to how to improve my own career from where it’s at the moment” [Mentee K].

### Post-Mentoring Interviews

#### A Confidence Boost for Many

Mentees and mentors felt that taking part in the mentoring programme built their confidence in several ways. One aspect of receiving mentorship that mentees found particularly helpful was how it encouraged them to *not define success by other people’s standards*: “To value myself because I’m me rather than place the values of others on myself if that makes sense, so stop like … to just say that I’m enough kind of thing” [Mentee B]. Both mentors and mentees commented on how the mentoring provided a space where mentors could give feedback to mentees on their work and *reflect on their achievements together*:

Being able to remind me about how effective these efforts I’m doing currently because with a lot of this kind of work you’re sort of shouting into a vacuum and you don’t get much feedback until something clicks and so to be told, or at least to sort of realise that the stuff that you’re doing is actually proactive and positive is a helpful step in itself [Mentee M].

Some mentees also felt that through their increased confidence, this mentorship had *opened the door to new opportunities*: “I’ve started to network and I’m like confident enough to go on my own and everything, which was a goal” [Mentee N].

#### Fostering an Empathetic Space

The mentoring programme was felt to provide a space to share experiences and the opportunity to seek advice. Both mentees and mentors commented on how their mentoring sessions had felt like *safe and supportive environments*, where they were not only able to share positive news but also tackle challenges and be supported through difficulty: “It seemed like there was a space that he could actually be really truthful about the things that he does actually genuinely struggle with” [Mentor S]. Mentees also commented on how they had enjoyed the fact that the mentoring programme had provided regular contact with another performing arts professional so that they *felt less alone* in the industry, and this had helped to normalise some of the challenges they faced: “It’s been reassuring really, you know, just knowing that… knowing that I’m not necessarily alone in my struggles” [Mentee K]. The mentees who had worked with autistic mentors unanimously reported that this had been a really positive aspect of their mentoring. Having *a mentor with lived experience of disability was highly valuable* because they had often shared similar challenges in their own professional lives and so were able to easily relate to difficulties faced by the mentees and offer advice based on their own experiences: “A benefit of having an autist mentor: they’d been through it and understood and had dealt with all that stuff themselves” [Mentee E]. The mentors expressed a desire to be able to share their experiences more widely and that *the knowledge exchange could go further*. They suggested that in future schemes it would be valuable to have opportunities for mentors to meet each other and exchange information and experiences: “I think it might have been useful to have a chat sort of halfway through the mentoring sessions with other mentors just to see how they’re managing that balance” [Mentor V].

#### A Mutual Learning Opportunity

Mentees and mentors ended the mentoring programme feeling like they had *gained new knowledge* and learnt or improved their skills through learning from each other: “Time management and organisation: with the things that we’d spoken about and the techniques that had been shared with me I thought, ‘I’ve got a better understanding of this now’” [Mentee F]. Mentees and their non-autistic mentors recognised that this programme had been a good learning opportunity for the non-autistic mentors to gain *increased autism knowledge*, which would be knowledge to take forward in their professional lives:

They’re not an expert in autism so I think the benefits that they got is that they spoke to an actually autistic artist… So, in terms of education about autism I think that was very good because now they can go away and they’ll go, ‘Oh yeah, I understand a bit more now about autism and that it’s a spectrum’ [Mentee G].

Mentees felt that taking part in the mentoring programme had been *a constructive experience* that that had involved positive professional development, and for some, it was transformative:

It’s just been really great to have this over this period of time. It’s got me focusing on really positive things, I’ve learned a lot from it, a huge amount. So yeah, I mean for me it’s been a very positive experience [Mentee J]

#### Good Communication is Key for Managing Expectations

Mentees highlighted the importance of effective communication, which meant they were on the same page with their mentors in terms of expected goals. It also helped to manage any anxiety the mentees had around previously unclear or unpredictable situations:

I think it worked well in terms of communication because it was always very, you know, we’ll speak on Skype on this day at this time and I knew what to expect and it was structured so we knew what we were going to be talking about and what the goals were so having the goals and the structure made me able to kind of follow the process if you know what I mean without getting anxious [Mentee N].

Some occasional breakdowns in communication did occur, however, which led to frustration and discord. One mentee stressed how important it was to:

Be a bit more aware of how much is being put on each other’s plate and enforce that only so many things should be discussed… be clearer with each other about how much communication is going to be had because the mentor was trying to get more out of me than I was able to give both in time and mental health wise [Mentee C].

#### The Relationship Can Make or Break the Support

The strength of the relationships between the mentors and mentees varied between partnerships. There were some *clashes of personalities* where perhaps the mentees and mentors were not well matched, and this led to some difficulties with communication and goal setting:

This phase started off a little bit more challenging just because of personalities as in mind-sets. It was a little bit more of a challenge than in the last [mentoring relationship] to begin with, but I think the results speak for themselves [Mentor Q].

There were also partnerships that worked exceptionally well, with mentees and mentors reporting that they had really got along with each other and these *strong bonds led to success*: “I felt like I built up a really good relationship with [my mentee] and we had a lot to talk about” [Mentor T].

#### A Need for Flexibility

The mentees and the mentors had a variety of preferences for how the mentoring was conducted, including many contrasting suggestions as to what worked well or did not across the programme. It was clear that *there was no one-size-fits-all approach*, and that support that was accessible to some did not work well for others. For example, some found the online method of speaking with their mentor practical:

I honestly think the video chat was probably best because meeting face-to-face would’ve caused so many sensory difficulties and so much exhaustion from doing that I wouldn’t have got the same out of it. So, it was actually really convenient [Mentee E]

Yet others struggled with the online format: “I think the fact we were only able to communicate over Skype or phone was a problem. I think it’s different whenever you’re with someone in person” [Mentee A]. Some mentees also wished that they could have taken part in the mentoring scheme across a different time period—and their mentors also recognised that it was important that *support needs to be implemented at the right time*: “I think it was a good idea in principle. I think [my mentee] and I had difficulties in that they just weren’t ready to plan or to work on anything, so that was kind of hard” [Mentor U]. A few mentors also struggled with the sometimes-needed flexibility around appointments that autistic people can require due to challenges predicting their future energy levels to cope with activities in advance, which sometimes led to last-minute cancellations. They spoke of how being accommodating can be challenging:

I’m really up for being flexible… like I think I really inconvenienced myself a few times because I was trying to just work with their schedule, so I think I probably could’ve been a bit more, ‘Yeah, we can rearrange but I can do this time’, rather than, ‘Yeah, sure, I can do four o’clock; I’ll make it work, kind of thing [Mentor T].

## Discussion

Autistic performing arts professionals report facing many work-related challenges (Buckley et al., 2020). Here, we examined whether professional mentoring might be one way to mitigate some of these challenges. Specifically, we tested the effects of a 10-week mentoring programme within the context of a pilot randomised controlled trial, directly measuring mentees’ occupational self-efficacy as well as eliciting their views and experiences using qualitative methods. We found that the programme had positive effects on both mentees and mentors, especially with regard to perceived gains in mentees’ occupational self-efficacy.

Feeling alone in the performing arts industry is a sentiment that has been reported by many performing arts professionals, who often feel that there is little support available to mitigate this isolation (Buckley et al., 2021). The mentees who received mentoring from a mentor who was also on the autistic spectrum found this shared identity a highly valuable aspect of the mentorship. Knowing that their mentor had already faced similar challenges reportedly allowed mentees to build a deeper relationship with their mentor and also receive more tailored advice on how to approach difficulties. This ‘meeting of (autistic) minds’ accords with research emphasising the importance of mutual understanding (Crompton et al., 2020; Milton, 2012). It also echoes research conducted by O’Mally and Antonelli (2016) in which legally blind students reported that being mentored by others with visual impairment, and thus being able to share common experiences and challenges, helped to boost their self-efficacy and engendered high satisfaction with their mentorship.

Importantly, however, these positive sentiments went beyond those mentees who were partnered with an autistic mentor. Mentees and mentors alike recognised the value of the mentoring sessions as a rare opportunity to speak openly and share experiences with like-minded, creative individuals. Critically, our mentees described the positive effects of being able to reflect upon work-related challenges and achievements, and to be encouraged and guided by their mentors, who had often also experienced such challenges and achievement. They also reported the boosts in self-confidence they experienced as a result. These qualitative reports were corroborated by our quantitative findings in which two thirds of the modification group mentees reported significant gains in occupational self-efficacy immediately after having taken part in the mentoring programme. Taken together, these findings suggest that mentoring might have a positive effect on mentees’ occupational self-efficacy, just as social-cognitive theory suggests (Bandura, 1986; see also St-Jean & Mathieu, 2015).

The relationship that forms between a mentor and mentee is critical, and pairings where mentees feel listened to and well supported are more successful in improving skills than those that are not (Roberts & Birmingham, 2017). Good and clear communication played a large part in the strength of the relationship for many of the mentees and mentors and several reported how it was helpful for managing expectations. This finding reflects previous research demonstrating that communicating clearly, particularly around boundaries, is beneficial for ensuring mentorship is successful and goals and appropriate behaviour are clearly understood by both mentee and mentor (Dawkins et al., 2016).

Such positive gains in self-efficacy build on previous studies that have examined employment-focused mentoring for different groups and consistently found self-reported confidence to have improved as a result of their involvement in a mentoring programme (Butterworth et al., 2012; Dashper, 2018; Gander, 2013; Lindsay et al., 2012, 2016). They are also consistent with mentoring studies within the field of autism employment research. For example, in one previous study, autistic participants (employment field not specified) showed increased wellbeing after having received mentoring and in interviews described how they had gained confidence (Martin et al., 2017). In another study, autistic people reported higher self-efficacy when receiving individualised autism-specific support in their workplaces compared to those who do not (Lorenz et al., 2016).

It is noteworthy that gains in occupational self-efficacy were not universally reported, which may in part be due to our small sample of mentees (n = 13) who completed the modification or control measures. That said, our qualitative analysis also revealed that the strength of relationship (and thus ‘meeting of minds’; Milton, 2012) between mentee and mentor also varied between pairs, which may have influenced the extent and nature of the effects of the mentoring. Furthermore, the structure of the programme was not always well-suited for some. Indeed, while there was high variability in what each mentee and mentor liked and disliked about the structure of the mentoring programme, the need for flexibility within the specified structure was clear. From how the pairs communicated with each other, to being able to re-arrange sessions at short notice, many of the autistic mentees required an adaptive and responsive approach. Such flexibility has been recognised as an important aspect of mentoring for autistic people to ensure that mentees are able to consistently access the support (Dawkins et al., 2016; Ridout & Edmondson, 2017). This need for adaptation has implications for how best to implement future mentoring programmes, namely with as much flexibility into the design of programmes as possible because of the wide-ranging needs and preferences of the autistic population.

Broader discussions of mentoring emphasise that mentee-mentor relationships should be reciprocal in nature (Haggard et al., 2011). In this vein, and consistent with previous research (Hamilton et al., 2016; Martin et al., 2017; Remington & Pellicano, 2018), our non-autistic mentors reported the benefits of providing support in this way, including perceived gains in their knowledge of autism and working with autistic people. Encouragingly, mentors also described how they would use what they had learned and apply it to their own practice and companies, meaning that this programme may provide extended benefits to wider employment practices among those who have been trained and worked as mentors. While little is known about precisely how non-autistic people might become more effective social supports for autistic people (cf. Crompton et al., 2020), mentoring might be one way to achieve this goal (Son & Kim, 2013; St-Jean & Mathieu, 2015).

## Limitations

This research is not without its limitations. First, this study examined the experiences of a small and selective sample of autistic performing arts professionals and mentors, which necessarily limits the generalisability of the findings. That said, the sample was diverse and well distributed in terms of gender, age, and career choices of the mentees. Second, the matching of the mentors and mentees was not blind, which may have unduly influenced the results. Instead, matching was based on shared areas of interest and experience in order to maximise the potential benefit to mentees of receiving mentorship from a mentor with experience they considered relevant to their interests and who was able to offer advice concerning a career path they may wish to pursue. This procedure therefore may be more likely to capture everyday mentoring practice.

## Conclusions

In sum, this study presents the results of an initial trial of a mentoring programme for autistic performing arts professionals. We found strong qualitative evidence that the mentoring programme was well received and felt to be beneficial by the participating mentees and mentors, particularly with regard to gains in mentees’ occupational self-efficacy. Developing strong, trusting mentor–mentee relationships within a mentoring programme that is sufficiently responsive to autistic mentees’ needs and preferences is an important avenue for future research and practice.
